# All-Cause Mortality Risk in Australian Women with Impaired Fasting Glucose and Diabetes

**DOI:** 10.1155/2017/2042980

**Published:** 2017-06-18

**Authors:** Lelia L. F. de Abreu, Kara L. Holloway, Mohammadreza Mohebbi, Muhammad A. Sajjad, Mark A. Kotowicz, Julie A. Pasco

**Affiliations:** ^1^Deakin University, Geelong, VIC 3220, Australia; ^2^Department of Medicine-Western Health, Melbourne Medical School, The University of Melbourne, St Albans, VIC 3010, Australia; ^3^Barwon Health, Geelong, VIC 3220, Australia

## Abstract

**Aims:**

Impaired fasting glucose (IFG) and diabetes are increasing in prevalence worldwide and lead to serious health problems. The aim of this longitudinal study was to investigate the association between impaired fasting glucose or diabetes and mortality over a 10-year period in Australian women.

**Methods:**

This study included 1167 women (ages 20–94 yr) enrolled in the Geelong Osteoporosis Study. Hazard ratios for all-cause mortality in diabetes, IFG, and normoglycaemia were calculated using a Cox proportional hazards model.

**Results:**

Women with diabetes were older and had higher measures of adiposity, LDL cholesterol, and triglycerides compared to the IFG and normoglycaemia groups (all *p* < 0.001). Mortality rate was greater in women with diabetes compared to both the IFG and normoglycaemia groups (HR 1.8; 95% CI 1.3–2.7). Mortality was not different in women with IFG compared to those with normoglycaemia (HR 1.0; 95% CI 0.7–1.4).

**Conclusions:**

This study reports an association between diabetes and all-cause mortality. However, no association was detected between IFG and all-cause mortality. We also showed that mortality in Australian women with diabetes continues to be elevated and women with IFG are a valuable target for prevention of premature mortality associated with diabetes.

## 1. Introduction

Impaired fasting glucose (IFG), a precursor of diabetes, is increasing in prevalence and has been associated with increased risk of cardiovascular disease [1–8]. IFG is defined by the American Diabetes Association (ADA) as elevated fasting plasma glucose (FPG) and insulin resistance. IFG is characterised as a FPG level between 5.5 and 6.9 mmol/L (100–125 mg/dL) without antihyperglycaemic medication, whereas diabetes is classified by FPG ≥ 7.0 mmol/L (126 mg/dL) [9]. We have recently reported that 33.8% of Australian women have IFG and the likelihood of progressing to diabetes over the ensuing decade was almost sixfold greater if FPG ≥ 6.1 mmol/L [10]. Diabetes mellitus is a complex disease that is also increasing in prevalence and can lead to serious health complications, such as nephropathy [11], retinopathy [12], neuropathy [13], stroke [11], coronary artery disease [2], lower limb amputation [14], and early mortality [15, 16]. It is estimated that 382 million people worldwide have diabetes, and this number is expected to rise to 592 million by 2035 [17]. In addition, it has been predicted that diabetes is responsible for 4 million deaths worldwide in 2010 [15]. In Australia, diabetes is a growing disease with 280 new cases diagnosed every day [18].

Diabetes is associated with premature mortality [17], but whether IFG is also associated with mortality risk is controversial. Some studies have demonstrated a relationship between IFG and mortality [1, 2]; whereas others have reported no associations [19–21]. Therefore, the aim of this study was to determine whether there is an association between IFG or diabetes and mortality in Australian women followed prospectively over a 10-year period.

## 2. Methods

### 2.1. Study Design and Subjects

This study uses data from the Geelong Osteoporosis Study (GOS), a population-based study including participants residing in the Barwon Statistical Division. This region is situated in southeastern Australia and has a large, stable population of approximately 280,000 and is largely representative of the Australian population, making it ideal for epidemiological studies. The region also contains residents with a range of cultural and socioeconomic characteristics; however, most participants were of European ancestry. A complete description of the methodology has been published elsewhere [22]. At baseline (1993–1997), an age-stratified random sample of 1494 women aged 20–94 years was recruited from Commonwealth electoral rolls, with a participation of 77.1%. We excluded 326 women because we did not have a FPG level or self-report of antihyperglycaemic medication or diabetes status, leaving 1167 eligible women for this analysis. Those who were excluded were older, shorter, and had lower weight, lower lean mass, greater waist circumference, higher serum triglycerides, lower serum HDL cholesterol, higher systolic and diastolic blood pressure, with a lower proportion of smokers, and lower mobility.

All-cause mortality was collated through the National Death Index. The study was approved by the Barwon Health Human Research Ethics Committee, and written informed consent was obtained from all subjects.

### 2.2. Measurements

All exposure measurements were performed at baseline. Weight and height were measured to the nearest ±0.1 kg and ±0.1 cm, and body mass index (BMI) was calculated as weight/height^2^ (kg/m^2^). Subjects were categorised as obese if BMI ≥ 30.0 kg/m^2^ [23]. Waist circumference (minimal abdominal, between the ribs and iliac crest) and hip circumference (maximal gluteal position) were measured to the nearest ±0.5 cm. Waist-to-hip ratio (WHR) and waist-to-height ratio (WHtR) were calculated from these measurements. Whole-body scans were performed using a dual-energy X-ray absorptiometry (DXA; Lunar DPX-L; Lunar, Madison, WI). These scans also provided estimates of body fat mass (kg), percentage body fat (%BF), and “lean” mass (kg), which includes the muscle, skin, connective tissue, and the lean component of adipose tissue (water and protein). We used a cut point of %BF > 30 for obesity [24]. Blood pressure was measured in a sitting position using an automated device (Takeda Medical UA-751). Women were considered to be hypertensive if they had a systolic blood pressure over 140 mmHg and/or a diastolic pressure above 90 mmHg and/or use of antihypertensive medication. Physical activity, alcohol consumption, current smoking, and medication use were self-reported by a questionnaire. Women who reported undertaking regular physical activity were described as active; otherwise, they were classified as inactive; high alcohol consumption was recognised if alcohol was consumed at least several times a week.

Venous blood was collected at baseline after an overnight fast. Fasting glucose was measured using an adaptation of the hexokinase-glucose-6-phosphate dehydrogenase method [25]. Blood samples were collected in sodium fluoride tubes by the major pathology centre in the region, and glucose assessment was completed soon after the blood collection. There was no long-term storage of blood samples before measurements. Diabetes was classified if FPG ≥ 7.0 mmol/L (126 mg/dL), self-reporting diabetes, and/or use of antihyperglycaemic agents (antihyperglycaemic medication use referred to medications taken regularly and currently at baseline). IFG was considered present if FPG level was between 5.5 and 6.9 mmol/L (100–125 mg/dL); according to the 2003 ADA diagnostic criteria [9]. Approximately, half (51.9%) of diabetes cases were diagnosed using multiple criteria including FPG and self-report (13%), self-report and medication (24.6%), and glucose, self-report, and medication use (14.3%). Only 22% and 26% of diabetes cases were diagnosed from glucose and self-report, respectively. Commercially available kits and clinical chemistry analyser (Thermo Fisher Scientific) were used to determine total cholesterol, high-density lipoprotein cholesterol (HDL-C), low-density lipoprotein cholesterol (LDL-C), and triglycerides. The use of lipid-lowering medications was investigated, but few women used these agents (*n* = 51). In these women, serum lipid results were still outside the range recommended by the World Health Organization (triglyceride < 2.0 mmol/L; HDL level > 1.29 mmol/L; LDL level ≤ 3.5 mmol/L) [26]. We also determined whether the participants had metabolic syndrome based on the International Diabetes Federation (IDF) criteria [27], which included measurements of serum triglycerides, serum HDL, waist circumference, and hypertension. FPG was excluded in this analysis since our data were presented in groups based on normoglycaemia, IFG, and diabetes. Succinctly, if a participant had a waist circumference > 80 cm and had at least two of the following: (i) raised TG level: ≥1.7 mmol/L, or specific treatment for this lipid abnormality; (ii) reduced HDL cholesterol: <1.29 mmol/L, or specific treatment for this lipid abnormality; (iii) raised blood pressure: systolic BP ≥ 130 mmHg or diastolic BP ≥ 85 mmHg, or treatment of previously diagnosed hypertension; and then a participant was considered to have metabolic syndrome.

### 2.3. Statistical Analyses

Kruskal-Wallis test for continuous data was used to compare subject characteristics between the three glycaemia groups (normoglycaemia, IFG, and diabetes). Categorical data were compared using the chi-square test (or Fisher's exact test). Overall survival rate per glycaemia groups was calculated using Kaplan-Meier product limit estimator. Survival curves were calculated accordingly. Survival rates were compared between the three groups with the use of a two-sided log-rank test. The hazard ratios (HRs) for the diabetes group and IFG, as compared with the normoglycaemia group, and corresponding 95% confidence intervals (95% CI) were estimated with the use of a Cox proportional hazards model. We followed the participants from their baseline appointment (1993–1997), until 31 of December 2012. We assessed a prespecified set of baseline characteristics for their relevance as prognostic factors of overall survival. We used a Cox proportional hazards model, including diabetes status stratum, to perform bivariate analysis of overall survival. Baseline characteristics significant at a 0.1 level were then used to construct the multivariable model. We implemented a backward elimination process with inclusion criteria of entry *p* value < 0.1 and exit *p* value > 0.05 to identify the final model. We explored two-way interactions of dichotomised diabetes status (diabetes versus nondiabetes) with all other significant factors in the final model. Due to data sparseness interaction with 3-level diabetes status was not possible. Estimated HR and two-sided 95% CI and *p* values were calculated for relevant prognostic factors. SPSS 22 was used for data analysis.

## 3. Results

At baseline, there were 696 (59.6%) women with normoglycaemia, 395 (33.8%) with IFG, and 76 (6.5%) with diabetes. The descriptive statistics for these women are presented in Table 1.

Women with diabetes were older than those with impaired fasting glucose and those with normoglycaemia. In addition, those with diabetes had higher measures of adiposity than those with impaired fasting glucose or normoglycaemia (*p* < 0.001). Measures of blood pressure were higher in those with impaired fasting glucose and diabetes compared with the normoglycaemic group. Serum triglycerides and LDL cholesterol were higher in the IFG and diabetes groups compared with the normoglycaemic group, whereas HDL was lower in those with impaired fasting glucose or diabetes. More women with IFG (39.0%) or diabetes (68.4%) had metabolic syndrome than women with normoglycaemia (21.6%). Unadjusted mortality was higher in those with diabetes (*n* = 41; 53.3%) than those with impaired fasting glucose (*n* = 89; 22.5%) and those with normoglycaemia (*n* = 87; 12.5%).

**Figure 1:**
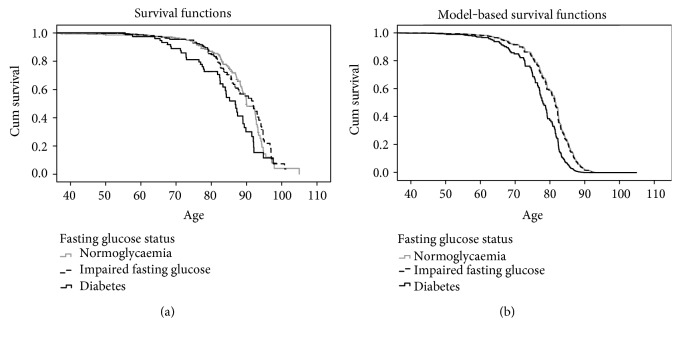
Unadjusted (a) and model adjusted (b) cumulative survival functions for fasting glucose status versus age at death (*x*-axis). Adjusted model includes fasting plasma glucose, age at cohort entry, and smoking status.

Bivariate analyses showed the following baseline characteristics to be candidate prognostic factors for overall survival: diabetes status, age, serum LDL level, and smoking (Table 2). Kaplan-Meier estimates of overall survival by diabetes status are shown in Figure 1. Those with IFG had a similar overall survival to women with normoglycaemia over the follow-up period, but individuals with diabetes had a shorter survival time compared to the other two groups.

Table 3 also shows the final adjusted model for the analysis of diabetes status and mortality. After adjustment for age and smoking status, the mortality rate for those with IFG was not different to the normoglycaemia group (HR 1.0; 95% CI 0.74, 1.4), but those with diabetes showed a higher mortality (HR 1.8; 95% CI 1.3, 2.7). Smoking was independently associated with an increased mortality with a hazard ratio of 2.0 (95% CI 1.3, 3.1). Additionally, age at cohort entry was a significant predictor of mortality, with a hazard ratio per year of age of 0.93 (95% CI 0.91, 0.95). Model-adjusted cumulative survival curves by diabetes status are shown in Figure 1.

Interactions between the variables in the model were investigated. Only one interaction term was significant: diabetes status and age (HR 0.964; 95% CI 0.934, 0.996; *p* = 0.025). This interaction shows that with increasing age, the risk of mortality associated with diabetes decreases.

## 4. Discussion

This longitudinal study determined the association between IFG and diabetes and all-cause mortality over a 10-year period in Australian women. Individuals with diabetes were older and had higher indices of adiposity, serum triglycerides, serum LDL cholesterol, blood pressure, alcohol consumption, and more physical inactivity compared to the other groups. The diabetes group also had lower serum HDL cholesterol compared with the IFG and normoglycaemia groups. All-cause mortality was greater in those with diabetes, whereas we did not detect a difference in the mortality risk for IFG compared to the normoglycaemia group. In addition, smoking and age were independent predictors of all-cause mortality in the final adjusted model. We found that older age entry at cohort was associated with a reduced all-cause mortality, which may be explained by a “healthier participant bias” [28]. Individuals in older age groups who were alive and agreed to participate in our study at baseline were likely to be healthier than those who declined to participate. This may have created a bias for mortality in the older age group. This study also showed that the association between diabetes and mortality was independent of metabolic syndrome. This reinforces the importance a tight glycaemia control and that elevated FPG in the diabetes range increases the risk of mortality [29]. Recent data from the United States [30] indicates that mortality attributed to diabetes is up to three times higher than death certificate data would suggest, with many cases of diabetes-related mortality being classified as cardiovascular disease, renal failure, or sepsis, leading to an underestimate of the contribution of diabetes to all-cause mortality.

There have been many studies investigating the association of IFG with mortality which have been collated in a meta-analysis which included 26 articles [31]. Using ADA criteria for IFG, the meta-analysis reported that IFG was not associated with an increased risk of all-cause mortality, compared to normoglycaemia (RR 1.07 95% CI 0.92–1.26), similar to our results. Other additional studies did not find any association between IFG and mortality risk [19–21]. Among US participants aged 52–75 years followed for 6.3 years, there was no association between IFG (ADA criteria) and all-cause mortality (HR 0.93; 95% CI 0.70–1.24) [19]. Two additional US studies with follow-up periods of 7.5 [1] and 13 years [21] and a Finnish study with 13 years of follow-up did not demonstrate an association between IFG and all-cause mortality [20].

Other studies have reported an association between IFG and mortality. For example, a prospective study of 2641 middle-aged men from Eastern Finland followed over 19 years showed that IFG was a risk factor for all-cause mortality (relative risk (RR) 1.31 (95% CI) (1.11–2.87)) [2]. Our study had a similar follow-up time; however, we did not show an association between IFG and all-cause mortality. One reason for this may be due to sample size; the Finnish study had more participants who could have their FPG followed. Although that study did not report the RR of FPG for all-cause mortality, they did show that there was a 10% increase in risk of sudden cardiac death with a 1.0 mmol/L increase in FPG (RR 1.10 (95% CI) 1.04–1.20, *p* = 0.001). Other important differences may be the longer duration of the Finnish study compared to our study, and that we included only women, whereas the Finnish study included only men.

By contrast, many studies have shown a clear association between diabetes and all-cause mortality. A study including data from 22 European cohorts investigated the association between diabetes (FPG ≥ 7.0 mmol/L) and all-cause mortality. Diabetes was associated with an increase in all-cause mortality across an 11-year follow-up; data combined from all 22 cohorts resulted in a HR of 1.6 (95% CI 1.4–1.8), similar to our estimate (HR 1.8, 95% CI 1.26–2.7) [32]. Some studies have investigated all-cause mortality in type 2 diabetes patients and consistently reported that individuals with diabetes have a higher risk of all-cause mortality, similar to our results [33, 34]. Saydah et al. have also estimated that diabetes is responsible for 3.6% of all deaths in Americans adults [35].

Diabetes often develops in the context of a westernised lifestyle, poor diet, and low-physical activity, leading to increased obesity levels and, eventually, beta-cell failure and diabetes. Additionally, systemic inflammation, hypertension, and dyslipidaemia occur simultaneously which leads to cardiovascular disease and increased risk of mortality [29]. In our study, we observed many of these lifestyle and physiological risk factors including lower physical activity and higher levels of obesity in women with diabetes compared to both IFG and normoglycaemia. Women with diabetes also had higher levels of hypertension and dyslipidaemia; 51.5%, 77.9%, and 25.0% had triglycerides, HDL and LDL, respectively, outside the recommended range.

Our study has some strengths and limitations. A major strength is that the participants were randomly selected and thus are representative of the general population. Our study also included a wide age range with considerable follow-up time. We also used a robust method for diabetes diagnosis, which combined a FPG measurement, self-report, and medication use. The study also utilised whole-body densitometry for the assessment of body fat mass and lean mass which more accurately assesses body composition estimated from anthropometric measurements [36]. Another strength of this study was complete ascertainment of mortality that was identified through the National Death Index. However, we acknowledge that there are some limitations to our study. The majority of the participants were white females, and our results may not be generalisable to other populations. The women who participated at baseline but who were excluded from the study due to insufficient information to classify diabetes status differed from those who were included in the study on factors that might have predisposed them to impaired fasting glucose or diabetes. Another limitation of the study was that diabetes status categorised based on a single FPG, whereas international guidelines require an elevated fasting glucose on at least two occasions. Furthermore, we did not have information on specific causes of mortality. Finally, we did rely on some self-reported data such as medication use, smoking, alcohol consumption, and physical activity, which may not be accurate, but it is important to note that most of our analyses were based on biochemical and clinical measurements as well as mortality data from the National Death Index.

## 5. Conclusions

In this study, we report that diabetes was associated with a higher risk of all-cause mortality; however, there was no difference detected in risk between women with IFG and women with normoglycaemia over 10 years of follow-up. We also showed that mortality in Australian women with diabetes continues to be elevated, indicating that secondary prevention measures have not been effective at reducing premature mortality in this group. Given that we did not find an association between IFG and all-cause mortality, women with IFG are a valuable target for prevention of premature mortality associated with diabetes.

## Figures and Tables

**Table 1:** Descriptive characteristics of women at baseline stratified by glycaemic status (NFG = normal fasting glucose, IFG = impaired fasting glucose, and diabetes). Data are shown as median (interquartile range) or *n* (%)^∗^.

	NFG (*n* = 696)	IFG (*n* = 395)	Diabetes (*n* = 76)	*p* value
Fasting plasma glucose (mmol/L)^∗^	5.0 (4.8, 5.2)	5.7 (5.6, 5.9)	8.1 (6.6, 11.1)	<0.001
Age (years)	42.1 (31.1, 42.1)	56.8 (44, 64.4)	65.2 (59.8, 75.3)	<0.001
Weight (kg)	63.8 (57.3, 72.8)	69.5 (61.1, 80.8)	71.5 (60.8, 83.2)	<0.001
Height (cm)	161.8 (157.9, 166.1)	160.5 (156.5, 165.3)	157.0 (153.0, 161.8)	<0.001
BMI (kg/m^2^)	24.5 (22, 27.7)	26.9 (23.9, 31.4)	30.0 (25.6, 33.3)	<0.001
BMI ≥ 30 (kg/m^2^)	104 (14.9)	124 (31.4)	38 (49.4)	<0.001
Body fat %	36.2 (30.6, 42.4)	41.0 (36.0, 45.7)	41.3 (37.1, 45.9)	<0.001
Body fat % > 30%	534 (76.7)	363 (91.9)	66 (85.7)	<0.001
Waist circumference (cm)^∗^	79.0 (72.5, 87.4)	87.0 (78.6, 96.5)	96.4 (87.1, 104.6)	<0.001
Hip circumference (cm)^∗^	101.2 (96.2, 108.3)	106.3 (99.3, 106.3)	108.7 (98.9, 119.8)	<0.001
Waist-to-hip ratio^∗^	0.78 (0.74, 0.83)	0.82 (0.77, 0.86)	0.88 (0.84, 0.91)	<0.001
Waist-to-height ratio^∗^	0.49 (0.44, 0.55)	0.54 (0.49, 0.6)	0.62 (0.56, 0.67)	<0.001
Systolic blood pressure (mmHg)^∗^	114 (104, 128)	128 (115, 140)	141 (126.3, 163.5)	<0.001
Diastolic blood pressure (mmHg)^∗^	74 (66, 82)	79 (71, 86)	78.5 (73, 92.8)	<0.001
Hypertension (%)^∗^^¥^	184 (26.4)	181 (45.8)	62 (81.6)	<0.001
Body fat mass (kg)^∗^	23.0 (17.5, 30.3)	28.0 (22.1, 36.2)	29.5 (21, 37.1)	<0.001
Lean mass (kg)^∗^	38.4 (35.9, 41.3)	38.6 (35.4, 42.2)	38.7 (36.1, 41.9)	0.7
Serum triglycerides (mmol/L)^∗^	0.94 (0.69, 1.36)	1.26 (0.89, 1.71)	2.01 (1.38, 2.42)	<0.001
Serum HDL cholesterol (mmol/L)^∗^	1.23 (1.02, 1.49)	1.20 (0.95, 1.45)	0.98 (0.84, 1.26)	<0.001
Serum LDL cholesterol (mmol/L)^∗^	2.71 (2.20, 3.36)	3.13 (2.55, 3.92)	2.96 (2.23, 3.44)	<0.001
Current smoker	120 (17.1)	56 (14.2)	13 (17.1)	0.4
High alcohol consumption	115 (16.5)	89 (22.5)	6 (7.9)	0.003
Physically inactive	146 (21)	127 (32.2)	47 (61.8)	<0.001
Metabolic syndrome^∗^^†^ (%)	150 (21.6)	154 (39.0)	52 (68.4)	<0.001
Mortality^#^	87 (12.5)	89 (22.5)	41 (53.9)	<0.001
Person years of follow-up	42856.63	28333.58	6013.39	—

∗ indicates missing data: fasting plasma glucose and blood pressure *n* = 22; body fat and lean mass *n* = 8; percentage body fat *n* = 8; waist and hip circumference, waist-to-hip ratio, and waist-to-height ratio *n* = 13; serum triglycerides *n* = 63; serum HDL cholesterol *n* = 61; serum LDL cholesterol *n* = 59; metabolic syndrome *n* = 1. ¥ indicates that hypertensive (y/n) was considered if the participant had a systolic blood pressure > 140 mmHg and/or a diastolic pressure > 90 mmHg and/or use of antihypertensive medication. † indicates metabolic syndrome excluding fasting plasma glucose. # indicates unadjusted mortality.

**Table 2:** Bivariate analyses and the final multivariable cox model for diabetes and mortality. All variables were measured at baseline.

Factor	Bivariate	*p* value	Multivariate	*p* value
	HR (95% CI)		HR (95% CI)	
Diabetes (yes)	1.61 (1.11, 2.35)	0.013	1.84 (1.26, 2.70)	0.002
Age (yr)	0.93 (0.91, 0.95)	<0.001	0.93 (0.91, 0.95)	<0.001
Weight (kg)	1.00 (0.99, 1.01)	0.621	—	—
Height (cm)	1.02 (1.00, 1.05)	0.068	—	—
BMI (kg/m^2^)	1.00 (0.97, 1.03)	0.824	—	—
BMI > 30.0 kg/m^2^	1.17 (0.85, 1.63)	0.341	—	—
Body fat % > 30%	1.31 (0.89, 1.93)	0.175	—	—
Waist circumference (cm)	1.00 (0.99, 1.02)	0.784	—	—
Hip circumference (cm)	1.00 (0.98, 1.01)	0.774	—	—
Waist-to-hip-ratio	2.67 (0.28, 25.37)	0.394	—	—
Waist-to-height ratio	0.76 (0.10, 5.67)	0.788	—	—
Systolic blood pressure (mmHg)	1.00 (0.99, 1.01)	0.901	—	—
Diastolic blood pressure (mmHg)	1.00 (0.99, 1.01)	0.422	—	—
Hypertensive (y/n)	0.81 (0.62, 1.07)	0.136	—	—
Body fat mass (kg)	1.00 (1.00, 1.00)	0.510	—	—
Lean body mass (kg)	1.00 (1.00, 1.00)	0.068	—	—
Serum triglycerides (mmol/L)	1.11 (0.93, 1.33)	0.234	—	—
Serum HDL (mmol/L)	0.93 (0.64, 1.34)	0.689	—	—
Serum LDL (<2.6 mmol/L)	0.74 (0.54, 1.01)	0.061	0.79 (0.58, 1.10)	0.160
Smoking	2.56 (1.64, 4.00)	<0.001	1.91 (1.21, 3.02)	0.006
High alcohol consumption	0.83 (0.59, 1.17)	0.278	—	—
Low mobility^∗^	0.92 (0.69, 1.22)	0.552	—	—
Metabolic syndrome	0.89 (0.68, 1.17)	0.413	—	—

∗ indicates that mobility was assessed with an ordinal scale as well (from 1 to 7), but *p* = 0.774.

**Table 3:** Multivariable model for evaluating diabetes status and risk of mortality.

Factor	*p* value	HR	Lower 95% CI	Upper 95% CI	Median survival (95% CI)^∗^
Glycaemia status	0.003	—	—	—	90.7 (89.7, 91.8)
Normoglycaemia	—	—	—	—	90.0 (87.3, 92.7)
Impaired fasting glucose	—	1.002	0.743	1.351	91.7 (89.4, 94.1)
Diabetes	—	1.843	1.256	2.703	86.8 (83.4, 91.6)
Smoking (yes)	0.003	1.962	1.251	3.077	82.5 (80.7, 84.3)
Age at cohort entry (years)	<0.001	0.932	0.912	0.953	—

^∗^Overall median survival age (years).
